# Further Development of Polyepichlorohydrin Based Anion Exchange Membranes for Reverse Electrodialysis by Tuning Cast Solution Properties

**DOI:** 10.3390/membranes12121192

**Published:** 2022-11-26

**Authors:** Mine Eti, Aydın Cihanoğlu, Enver Güler, Lucia Gomez-Coma, Esra Altıok, Müşerref Arda, Inmaculada Ortiz, Nalan Kabay

**Affiliations:** 1 Department of Chemical Engineering, Faculty of Engineering, Ege University, 35100 İzmir, Turkey; 2 Department of Chemical Engineering, Atılım University, 06830 Ankara, Turkey; 3 Department of Chemical and Biomolecular Engineering, Universidad de Cantabria, Av. Los Castros 46, 39005 Santander, Spain; 4 Department of Chemistry, Faculty of Science, Ege University, 35100 İzmir, Turkey

**Keywords:** salinity gradient, blue energy, reverse electrodialysis, anion exchange membrane, polyepichlorohydrin

## Abstract

Recently, there have been several studies done regarding anion exchange membranes (AEMs) based on polyepichlorohydrin (PECH), an attractive polymer enabling safe membrane fabrication due to its inherent chloromethyl groups. However, there are still undiscovered properties of these membranes emerging from different compositions of cast solutions. Thus, it is vital to explore new membrane properties for sustainable energy generation by reverse electrodialysis (RED). In this study, the cast solution composition was easily tuned by varying the ratio of active polymer (i.e., blend ratio) and quaternary agent (i.e., excess diamine ratio) in the range of 1.07–2.00, and 1.00–4.00, respectively. The membrane synthesized with excess diamine ratio of 4.00 and blend ratio of 1.07 provided the best results in terms of ion exchange capacity, 3.47 mmol/g, with satisfactory conductive properties (area resistance: 2.4 Ω·cm^2^, electrical conductivity: 6.44 mS/cm) and high hydrophilicity. RED tests were performed by AEMs coupled with the commercially available Neosepta CMX cation exchange membrane (CEMs).

## 1. Introduction

The use of fossil fuels causes negative impacts such as greenhouse gas emissions and global warming. Therefore, the need for clean and sustainable energy resources is increasing. The main sustainable energy sources are solar, wind, biomass and hydro energy. Other energy sources such as tidal power, ocean wave power, and ocean thermal energy transformation are also available but they are limited [1]. The global potential of salinity gradient energy, so-called blue energy, is around 1.4–2.6 TW. Therefore, the blue energy potential is very important and valuable to be considered as an alternative and renewable energy source [2]. Salinity gradient energy can be obtained from the controlled mixing of two water masses of different salt concentrations. It can be used where the river meets the sea and is different from solar and wind energy because it is continual [3]. The theoretical energy that can be generated from mixing 1 m^3^ of river water (1 g NaCl L^−1^) with 1 m^3^ of seawater (30 g NaCl·L^−1^) is 1.7 MJ [4]. About 25% of CO_2_, 27% of CH_4_, and 8% of N_2_O emissions could be decreased by salinity gradient energy [3]. For producing salinity gradient energy, the chemical potential difference due to the concentration difference of two different salt solutions is used to generate electricity. It is not harmful to the environment because it does not produce carbon dioxide (CO_2_) and any other gases.

**Figure 1:**
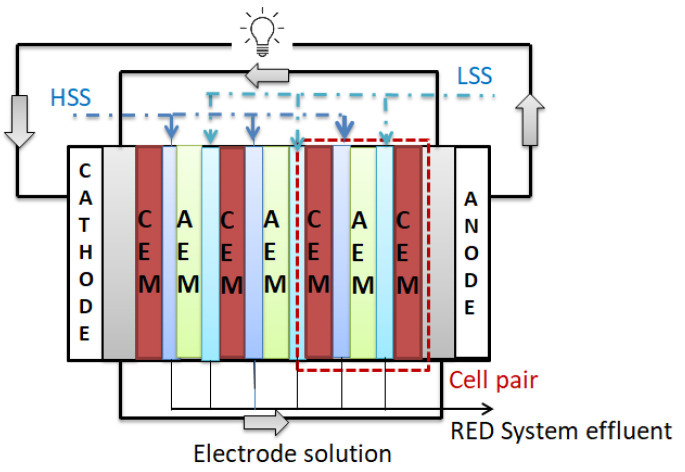
Schematic of a RED system (HSS: High saline solution, LSS: Low saline solution) (Adapted from [9]).

Among technologies that produce energy based on salinity gradient, the RED system is one of the most significant technology due to its production of a high-power density and functionality [5,6]. In the RED system, the membrane stack consists of successive arrangement of certain numbers of IEMs placed between electrodes. A cell pair in the RED stack consists of one CEM and one AEM. During RED operation, the salinity gradient on both sides of a membrane allows ions to move in the opposite directions (Figure 1). This ion migration creates a Nernst potential between cells, and this potentially triggers oxidation and reduction reactions in the anode and cathode, respectively. The redox processes in the electrodes induce by the Nernst potential difference. Redox couples in the electrode compartment, referred to as the “electrode solution”, play a crucial function in the system, as they convert the electrical current from the flow of electrons. Between cathode and anode, the electrode solution is constantly being exchanged. Electrons flow across an external circuit, resulting in an electrical current between the anode and cathode. Electricity is generated as a result of the flow of this current [7,8,9].

The commercialization of RED is reliant on low membrane cost and excellent power performance [10]. Most commercial AEMs are not designed specifically for RED. They are instead designed for various applications such as electrodialysis, diffusion dialysis, electro-deionization, and fuel cells [11]. Therefore, in recent years, many studies on ion exchange membrane synthesis for RED applications have been reported [7,11,12,13]. AEMs are essential parts of RED and have a significant impact on the overall performance of the process [14,15]. In AEMs, (NH_3_^+^), (NRH_2_^+^), (NR_2_H^+^), and (NR_3_^+^) are examples of positively charged functional groups that transport anions but not cations [16].

The most common polymers preferred in the synthesis of AEMs that use in RED applications are polyvinyl alcohol (PVA), poly(diallyl dimethylammonium chloride) (PDDA), polystyrene (PS), poly(2,6-dimethyl-1,4-phenylene oxide) (PPO), polyethylene (PE), poly(arylene ether sulfone) (PAES), poly(diallyl dimethylammonium chloride) (PDDA), and polymethyl pentene (PMP) [13,17,18,19,20,21,22,23,24,25,26]. These materials are attractive because of the chemical resilience, membrane processability and low cost.

Amination was the earliest and most reliable chemical process used to prepare common AEMs for RED [27]. Chloromethylation reaction is toxic and carcinogenic in the preparation of AEMs. Güler prepared safe and environmentally friendly AEMs for RED using polyepichlorohydrin (PECH) [28]. This procedure was followed with the addition of polyacrylonitrile (PAN) to increase the mechanical strength of the membrane with a blend ratio up to one. The amination process was performed on poly(vinyl alcohol-co-ethylene) (PVA-co-PE) [19], brominated poly(2,6-dimethyl-1,4-phenylene oxide) (PPO) [20] and poly(arylene ether sulfone) (PAES) [7] for the synthesis of AEMs for RED. Lopez et al. prepared a PECH-based AEM using a solution casting technique and the surface of the AEMs were modified with poly(ethylenimine) and glutaraldehyde for the structural modification [29].

There are still unexplored experimental conditions to further develop PECH-based AEMs. The quaternary agent and active polymer blend ratios are the most significant parameters affecting the physical (SD, IEC, FCD) and electrochemical properties (area resistance and electrical conductivity) of AEMs, resulting in the energy-harvesting capacity change of membranes. While the quaternization degree controls the ion exchange capacity, swelling degree, membrane resistance, etc., the active polymer blend ratios help to improve flexibility and crosslinking degree. To the best of our knowledge, thus far limited studies have investigated the effect of these two significant parameters [12,30]. Thus, the primary purpose of this study is to observe the effect of amination degree and active polymer blend ratios on the physical and electrochemical properties of AEMs for energy harvesting from a comprehensive perspective. To this end, in this study, the physical (SD, IEC, FCD,) and electrochemical (area resistance, electrical conductivity) properties of synthesized AEMs were investigated in detail by changing the quaternary ammonium agent and active polymer blend ratios in the range of 1.00–4.00 and 1.07–2.00, respectively, to find improved properties of the membranes.

## 2. Materials and Methods

### 2.1. Synthesis of AEMs

In this work, AEMs were prepared from a polymer solution by the casting method followed by solvent evaporation. For the synthesis of membranes, PECH (37 wt% chlorine, Osaka Soda Co., Ltd., Osaka, Japan) as the active membrane material and PAN (Mitsubishi Chemical Co., Ltd., Tokyo, Japan) as the inert polymer were used. To provide a good mechanical stability to the membranes, the crosslinker, 1,4-diazabicyclo[2.2.2]octane (DABCO, Reagent Plus ≥ 99%, Sigma-Aldrich, Burlington, MA, USA) with diamine functionality was used. The base membrane components were dissolved in dimethyl sulfoxide (DMSO) solution [28].

The active polymer solution was prepared by dissolving PECH in DMSO. The inert polymer solution was prepared by dissolving PAN in DMSO by stirring the mixture for 24 h at room temperature. The amination solution was obtained by dissolving DABCO in DMSO on a magnetic stirrer for 3 h. Then, the active and inert polymers and amination solutions were combined. This mixture was stirred in a flask at 80 °C using an oil bath for half an hour. The membrane casting solution was then prepared [28].

**Figure 2:**
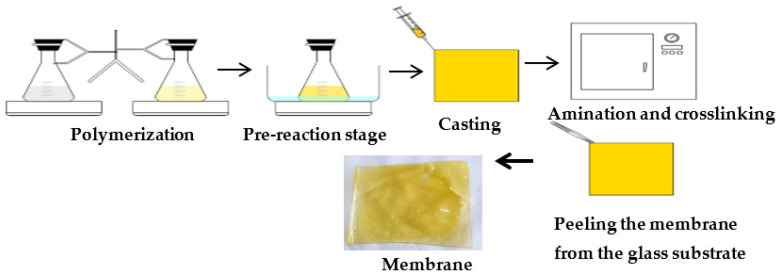
Preparation procedure of AEMs.

For the synthesis of small-scale AEMs (petri size, diameter: 55 mm) a certain amount of casting solution was transferred to the petri dishes with a syringe and the petri dishes were covered with glass lids in order to prevent the evaporation of DABCO during the amination process. Petri dishes were placed into an oven and a temperature of 110 °C was applied for 2 h under nitrogen gas atmosphere for completing the amination reaction and crosslinking formation. Then, the lids of the petri dishes were removed, and the samples were kept in the oven at 130 °C under nitrogen for half an hour in order to evaporate the remaining solution [28]. After the petri dishes were cooled down, the polymer films were separated from the glass surface and stored in 0.5 M NaCl. For RED tests, AEMs (15 cm × 15 cm) were synthesized with an excess diamine ratio of 4.00 and blend ratio of 1.07. The casting solution was transferred to the glass substrate with a syringe and the mold was covered with glass lids in order to prevent the evaporation of DABCO during the amination process. The preparation procedure of AEMs is shown in Figure 2.

Blend ratio (*σ*) is defined as the mass ratio of PECH to PAN and expresses the active polymer ratio per unit inert polymer by mass as explained in Equation (1):

(1) $$\sigma =\frac{m_{PECH}}{m_{PAN}}$$

DABCO is added to the casting solution for amination and cross-linking for PECH to react with chloromethyl groups to create the specific positive charge required for ion exchange. The excess diamine ratio (*ν*), shows the molar ratio of the amine component in DABCO (*m^d^*, mmol/g diamin) and chloromethyl groups in PECH solution (*m^p^*, mmol/g—CH_2_Cl). This expression also indicates how many chloromethyl groups are used in the amine (DABCO) amount as explained in Equation (2):

(2) $$\nu =\frac{m^{d}}{m^{p}}$$

AEMs were synthesized with different blend ratios (BR) and excess diamine ratios (ER) as explained in Table 1 to determine how these two parameters affect the membrane properties (SD, IEC, etc.) and their performance in the RED operation.

**Figure 3:**
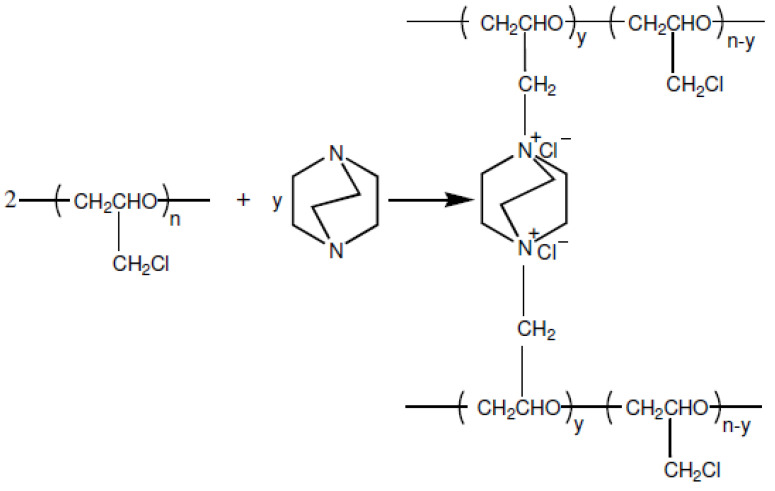
Reaction mechanism of amination-crosslinking of PECH polymers [12].

The amount of PAN to be added to the casting solution was varied to obtain different blend ratios of the membrane containing a certain amount of active, anion exchange polymer. The terms blend ratio and excess diamine ratio are used to determine the ratios of active polymer and diamine that will determine the fundamental properties of membranes such as IEC, SD and conductivity. Thus, the membrane properties based on these terms were determined. DABCO was added to the casting solution for amination and cross-linking for the reaction with chloromethyl groups of PECH to create the positively charged fixed groups required for ion exchange (Figure 3).

### 2.2. Characterization of AEMs

#### 2.2.1. Scanning Electron Microscope (SEM)

The cross-section morphology of the synthesized AEMs was determined using SEM analysis. Before the analysis, the membrane samples were dried at 30 °C and covered with a thin layer of gold using the EMITECH K550X device. SEM analysis of membrane samples was performed using the THERMO SCIENTIFIC APREO S device.

#### 2.2.2. Fourier-Transform Infrared Spectroscopy (FTIR)

FTIR analysis was performed to determine the chemical structure of the membranes. To this end, a Perkin Elmer Spektrum 100 Fourier Transform Infrared Spectrometer was used. Measurements were made using the ATR (Attenuated Total Reflection) module.

#### 2.2.3. Ion Exchange Capacity

IEC, which expresses the amount of charged groups in IEMs, was determined by the titration method. The AEMs were brought to the Cl^−^ ionic form by contact with a 3 M NaCl solution for 15 h. Then, the membrane was rinsed with ultrapure water to remove the excess amount of NaCl from the membrane surface. Whether the final rinse water contains Cl^−^ ions or not is checked using a 0.1 M AgNO_3_ solution. After rinsing the membranes with ultrapure water, the membranes were in contact with a 1.5 M Na_2_SO_4_ solution for 3 h to replace Cl^−^ ions in the membrane with SO_4_^2−^ ions in the solution. The solution containing released Cl^−^ ions was titrated with 0.1 M AgNO_3_ solution until the equivalence point. After that the AEMs were dried in a vacuum oven at 30 °C until they reached a constant weight and the *IEC* value (mmol/g-dry membrane) was calculated using Equation (3):

(3) $$IEC=\frac{V_{AgNO_{3}}}{w_{kuru}}\times C_{AgNO_{3}}$$

where $V_{AgNO_{3}}\;$indicates the volume of AgNO_3_ solution consumed in titration at the equivalence point, *w_dry_*, is the dry membrane weight, and $C_{AgNO_{3}}$ indicates the concentration of AgNO_3_ solution used.

#### 2.2.4. Swelling Degree

The SD indicates the water content of IEMs when exposed to water. To calculate the SD, the membranes were first removed from the salt solution, rinsed with ultrapure water and then kept in ultrapure water. After removing the membranes from water, their wet weight was measured, and the membranes were then dried in a vacuum oven at 30 °C until they reached constant weight. The *SD* (gH_2_O/gdry membrane) was calculated with the aid of Equation (4):

(4) $$SD\;\left(\%\right)=\frac{m_{wet}-m_{dry}}{m_{dry}}\times 100$$

where, *m_wet_* and *m_dry_* are the wet and dry weights of the membrane, respectively.

#### 2.2.5. Fixed Charge Density (FCD)

The ion transport of the membranes depends on the SD and the charged functional groups. This parameter, defined as the FCD, is preferred to be as high as possible. Constant charge density (*C_fix_*) (mmol/g-H_2_O), is expressed as the *IEC* per unit *SD* (mmol constant charge groups per g-membrane), and it is calculated by dividing the *IEC* by the *SD* (Equation (5)).

(5) $$C_{fix}=\frac{IEC}{SD}$$

#### 2.2.6. Electrical Conductivity and Area Resistance

Electrical power is generated from the potential difference arising from the mixing of two solutions with different salinities and can be recovered by the RED method. For this, the electrical resistance of the membrane stack should be as low as possible; in other words, the electrical conductivities of the IEMs in the stack should be as high as possible. IEMs with high electrical conductivity have high ion transport efficiency and therefore high electrical power generation potential. In this study, the electrical conductivity values were calculated from the results of the area resistance of the IEMs such that distance between the electrodes is divided by the product of resistance and area (width × thickness) of membrane samples. Membrane thickness values were measured with a digital micrometer (Mitutoyo, Japan). Area resistance measurements were performed with platinum electrodes using a Zahner Zennium potentiostat device and the electrochemical impedance spectroscopy method. Before the measurements, the AEMs were kept in an oven at 22 °C for 18 h.

### 2.3. RED Tests

**Figure 4:**
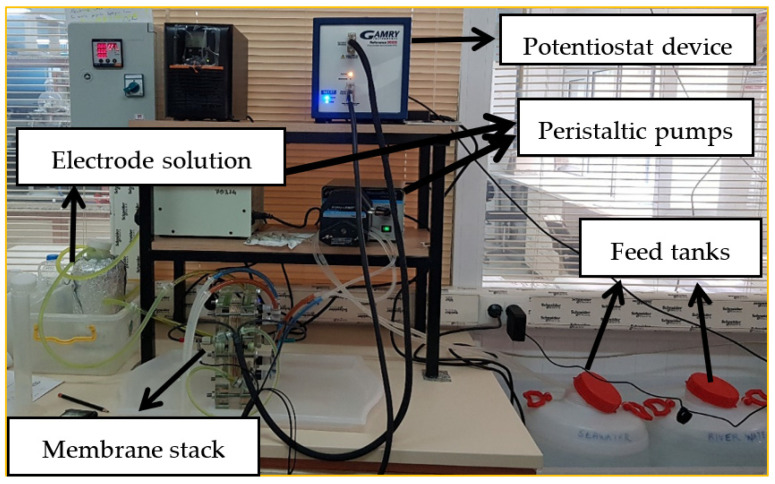
RED system components.

The RED system consists of a membrane stack, a potentiostat, two peristaltic pumps, an electrode solution, two feed tanks (low saline solution, LSS (1 g NaCl/L) and a high saline solution, HSS (30 NaCl/L)) (Figure 4). The membrane stack of the RED system consists of two end plates and electrodes (Ti-Ru alloy, 10 × 10 cm active area), CEMs and AEMs, and gaskets and spacers. The technical information about the RED system is given in Table 2. The characteristics of commercial NEOSEPTA CMX membranes are given in Table 3.

A mixture of 0.05 M K_4_Fe(CN)_6_, 0.05 M K_3_Fe(CN)_6_ and 0.25 M NaCl were used as the electrode solution and this solution was circulated between the electrode compartments. The ER4-BR1.07 AEMs were tested in the available RED system after casting AEMs in a proper size (15 cm × 15 cm). In the RED system tests, AEMs were used along with commercial Neosepta CMX. The current-voltage relations (I–V), current density and power density values were evaluated during the RED tests. The potentiostat device (Gamry Instruments Reference 3000) was used to monitor the electrochemical measurements in chronopotentiometric mode within a current density range of 0 A/m^2^ and 0.36 A/m^2^. The open circuit voltage (OCV) is defined as the maximum potential difference when there is no current and the circuit is not closed. The potential difference across a membrane can be determined by the Nernst equation as given in Equation (6) [20].

(6) $$E_{OCV}=N\frac{\alpha RT}{zF}\ln\left(\frac{a_{c}}{a_{d}}\right)$$

where *α* is the average membrane permselectivity of membrane pairs (-), *N* is the number of membrane cells (-), *R* is the gas constant (8.314 J/mol.K), *T* is the absolute temperature (K), z is the electrochemical valence, *F* is the Faraday constant (96485 C/mol), *a_c_* is the activity of the concentrated salt solution (mol/L), and *a_d_* is the activity of the diluted salt solution (mol/L). Increasing the concentration ratio ($\frac{a_{c}}{a_{d}}$) leads to a higher salinity gradient, which increases the voltage resulting in higher power output. The power generated (*W*) as Watt is found by multiplying the pre-determined current values (*I*) by the corresponding potential differences (*V*) using Equation (7). The power density is defined as the energy generated per membrane area (*A*), calculated by using Equation (8):

(7) W=V·I

(8) $$P=\frac{W}{2AN}$$

where *P* is the power density (W/m^2^), *W* is electrical power (W), *A* is active membrane area (m^2^), and *N* is the number of membranes. The RED test was carried out with the salinity ratio of 1:30 g/g and with the flowrates of feed solutions of 30 mL/min, 75 mL/min and 120 mL/min.

## 3. Results and Discussion

In the study of Karakoc and Guler, the effect of the blend ratio and the excess diamine ratio on SD and IEC were examined within the range of blend ratio between 0.6 and 2, and excess diamine ratio between 1 and 4 [30]. In a different work, Guler et al. studied the blending ratio and excess diamine ratio impact on SD and IEC within the diamine ratio between 2.6 and 7.3 and the blend ratio between 0.1 and 1 [12]. As a continuation of these studies, in this work, the ion exchange and swelling behavior were investigated by using blend ratios greater than 1 (1.07, 1.23, 1.50 and 2.00) and excess diamine ratios of 1, 1.62, 2 and 4, respectively. SEM and FTIR analyses were applied to the AEMs synthesized and physical characteristics (SD), and electrochemical properties (area resistance, electrical conductivity and IEC) were determined using standard methods.

### 3.1. SEM Analysis

**Figure 5:**
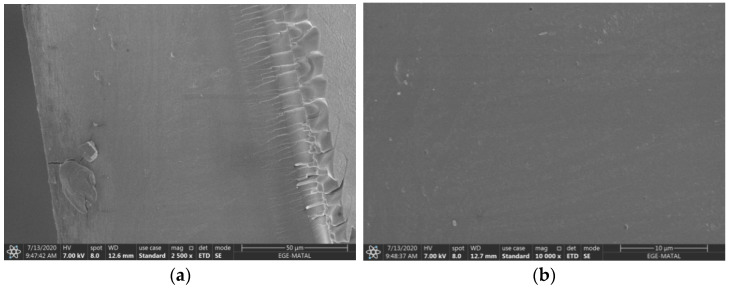
Cross-section SEM images of ER4-BR1.07 AEM (**a**) 2500×, (**b**) 10,000×.

SEM analysis was used to examine the cross-section morphology of prepared PECH-based AEMs. After applying the solvent evaporation process, cross-section SEM images of AEMs with excess diamine ratio (ν) 4 and blend ratio (σ) 1.07 at 2500× and 10,000× magnifications are shown in Figure 5.

In Figure 5a wrinkle-like structures were observed close to the air side of the membrane surface. These structures are irregularities due to solvent evaporation. When looking at the highly magnified images, it is understood that there are no physical pores in the membrane structure. The non-porous structure of the membranes indicates that the solvent evaporation method was successful. In Güler et al. (2014), a clear dense structure of the AEMs without pores can be recognized in a similar way [28]. Similarly, in Lopez et al. AEM presents a very homogeneous surface without pores [29].

### 3.2. FTIR Analysis

**Figure 6:**
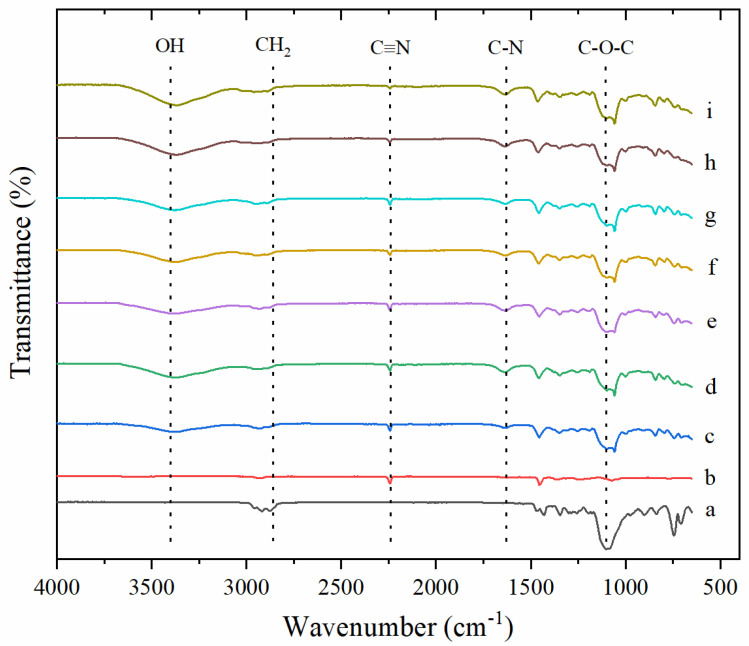
FTIR spectrums of samples (**a**) PECH (**b**) PAN (**c**) ER4-BR1.07 (**d**) ER2-BR1.07 (**e**) ER1.62-BR1.07 (**f**) ER1-BR1.07 (**g**) ER1.62-BR1.23 (**h**) ER1.62-BR1.5 (**i**) ER1.62-BR2.

FTIR analysis was performed to verify the reaction of PECH polymer chains with DABCO. The emergence of positive charges resulting from the reaction of chloromethyl groups in PECH polymers with the crosslinker DABCO is the quaternization process. FTIR spectra were illustrated in Figure 6. When PECH and PAN polymers were mixed, a peak was observed at 2240 cm^−1^ representing the nitrile group (−C≡N) of the inert polymer PAN, and the intensity of this peak declined with decreasing the blend ratio of PAN polymer as expected. For instance, the fingerprint peak intensity of the PAN polymer (2240 cm^−1^, ((−C≡N)) in the ER1.62-BR1.07 (peak e) membrane obtained much more than the ER1.62-BR2 (peak i) membrane, which show the preparation success of membranes. Similarly, after crosslinking PECH polymer with DABCO, a new peak was observed at 1640 cm^−1^ in the structure of crosslinked membranes, representing the C-N bond present in the DABCO structure [32]. This new peak proved the presence of quaternization in the membrane structure [30,33]. Intensity of this peak increased with the increase in blend ratio where active functional groups increase. Moreover, peaks at 2877 cm^−1^, 1452 cm^−1^ and 1108 cm^−1^ indicate the existence of CH_2_ and C-O-C groups in the PECH structure. As seen in Figure 6, the peaks observed at 1640 cm^−1^ and 2240 cm^−1^ indicate that the amination was carried out and thus it is clearly seen that the integration of PAN has been successful. The prominence of the peaks observed at 3400 cm^−1^ indicate the presence of free water molecules in the ion exchange membrane structure.

### 3.3. Properties of AEMs

**Figure 7:**
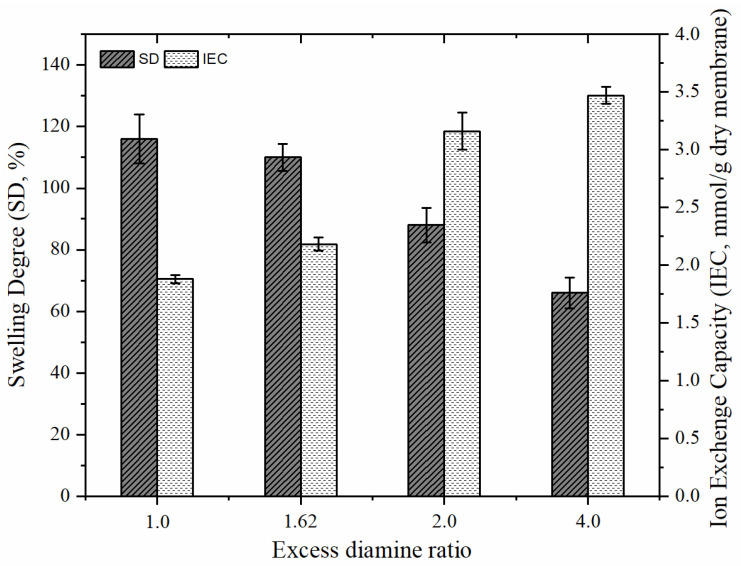
The effect of excess diamine ratio on SD and IEC of synthesized AEMs (BR:1.07).

**Figure 8:**
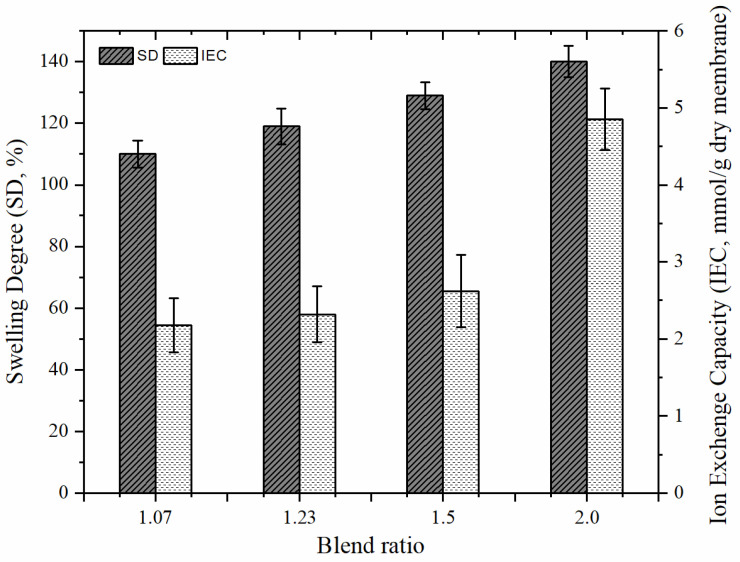
The effect of blend ratio on SD and IEC of synthesized AEMs (ER:1.62).

Different from the work by Güler et al. [28], we observed membrane properties for the diamine ratio and blend ratio values greater than one and examined the effect of the DABCO amount on membrane behavior. The effects of the excess diamine ratio and blend ratio can be seen in Figure 7 and Figure 8. As seen in Figure 7, as the blend ratio increases, SD increases up to 140%. This value is too high for AEMs in RED applications because excessive swelling is not adequate in terms of mechanical stability; therefore, the values of excess diamine ratio that affected the membrane properties (SD and IEC) were investigated as well in this study. It is expected that the crosslinking, and thus, the quaternization of the membrane will increase with the increase of the diamine ratio. Indeed, IEC of the membrane that contains relatively more charged functional groups increased (Figure 8). As DABCO also enables the formation of reticulated polymer chains (crosslinking), thus the SD of the AEMs will be controlled by the addition of DABCO (i.e., increasing diamine ratio).

As seen in Table 4, Güler et al. obtained the highest IEC value of 2.80 mmol/g dry membrane at a blend ratio of 1.04 and excess diamine ratio of 4.2. The SD value was determined as 120% at a blend ratio of 1.04 and at an excess diamine ratio of 4. According to Karakoc and Guler, the IEC value of the ER2-BR0.6 membrane was 2.8 mmol/g dry membrane while SD is 44% [30]. In this study, at an excess diamine ratio of 4 and blend ratio of 1.07 SD was 66%. In Lopez et al., SD of the membranes was 30.1% and the IEC value obtained as 1.4 mmol/g dry membrane [29]. Our results indicate that SD and IEC values are higher than the literature values because of higher excess diamine and blend ratios. At acceptable SD of the membranes, a relatively higher IEC is desired for the RED application to energy harvesting because ion transport through membranes is expected to be enhanced by a greater number of functional groups responsible for ion exchange.

Electrical conductivity values of AEMs synthesized in this study are given in Table 5. The highest conductivity value was obtained with the membrane with ER value of 4 and BR value of 1.07. Germer et al. reported the conductivity of commercial AEM (Nafion 212) as 3 mS/cm at 30 °C. They noted that the conductivity reached up to 8 mS/cm as the temperature increased [35]. Diaz and Kamcev determined the conductivity of AEMs with 152 µm of thickness as 7 mS/cm [34]. Tuan et al. measured the conductivity of the commercial AEM (AHA, Astom Corporation, Japan) as 4.5 mS/cm at 30 °C. That value increased up to 22 mS/cm in quaternized PECH membranes cross-linked with polyarylenether ketone [36]. Sarode examined the solvent and ion transport in AEMs under humidified conditions and obtained the membrane conductivity as 9 and 10 mS/cm at 30 °C and 95% relative humidity [37]. Pandey et al. found the electrical conductivity value as 7 mS/cm for the electron-beam grafted polyethylenetetrafluoroethylene-based AEMs at 30 °C and 95% of relative humidity [38], while Vandiver (2015) determined the electrical conductivity values as 4.8 ± 0.1 and 3.3 ± 0.2 mS/cm in AEMs (PFMA, methyl ammonium and PFTMBA, trimethylbenzyl ammonium) [39]. In this study, electrical conductivity values of the synthesized AEMs are in the range of literature findings.

The results of area resistance measurement of AEMs with different excess diamine and blend ratios are also given in Table 5. It is observed that the area resistances decreased as the diamine ratio and blend ratio increased because of the increased number of conductive sites created by diamine (DABCO) and active polymer (PECH) reaction. On the other hand, an increase in area resistance was observed in the membrane with a diamine ratio of 2, but it is insignificant, which is also acceptable for RED application. In Guler et al., AEMs prepared for the RED stack with a thickness of 77 µm, a SD of 32%, and an IEC value of 1.3 mmol/g-dry membrane was measured as 2.5 Ω·cm^2^ [12]. Rijnaarts et al. obtained an area resistance of 1.36 Ω·cm^2^ for AEMs (aliphatic Fuji V3B membrane) with a thickness of 84 µm, an IEC of 1.7 mmol/g-dry membrane, and a SD of 61% [40]. In a different study, the area resistance of AEMs (polystyrene/divinylbenzene/chloromethyl styrene AMV and APS membrane) with a thickness of 110–150 µm, a SD of 17%, and an IEC of 1.78 mmol/g-dry membrane is 2.8 Ω·cm^2^ [41]. In this study, the measured area resistances of AEMs were found to be higher than in the literature. It was considered that the differences in the membrane thickness, SD and IEC affected the area resistance results.

### 3.4. RED Performances of AEMs

The IEC and SD of large-scale and small-scale membranes were compared. The large scale (15cm × 15cm) ER4-BR1.07 membrane was selected for RED tests because it has optimum characteristics (low SD, high IEC, high FCD, etc.). Characterization of small (petri size diameter: 55 mm) and large scale (15cm × 15cm) AEMs is given in Table 6. The thickness of large-scale membrane is 250 ± 50 µm and the thickness of the small-scale membrane is 140 ± 20 µm. The SD value is very high for large scale membranes because of the thickness of the membrane and IEC values so close to each other.

**Figure 9:**
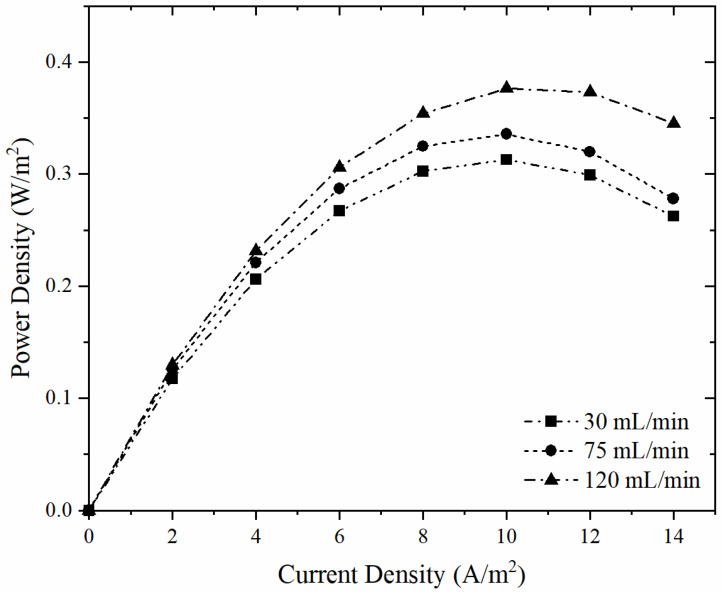
Power density values with ER4-BR1.07 and NEOSEPTA CMX membranes at different feed flowrates.

In RED tests, AEMs with 15 cm × 15 cm size were paired with commercial NEOSEPTA CEMs with the same size. Flow rates of feed solutions were 30 mL/min, 75 mL/min, and 120 mL/min. The number of membrane pairs was three and salinity ratio of dilute solution to concentrate solution was adjusted as 1:30 (g NaCl: g NaCl) for each study (Table 7). Power density vs. current density behaviour with ER4-BR1.07 and NEOSEPTA CMX membranes is shown in Figure 9. Power density values increased with increasing feed flow rate. Maximum power density value was obtained using the feed flow rate of 120 mL/min as 0.376 W/m^2^ due to the OCV. As the OCV increased, the power density values increased (Table 8).

Karakoc and Guler reported power density values with PECH-H, PECH-C and NEOSEPTA AMX membranes coupled with NEOSEPTA CMX membranes with the flow rates of 30, 60 90 and 120 mL/min. The best-performing membrane, PECH-C, which has a BR of 0.6 and an EDR of 2.0, can produce a power density of up to 0.32 W/m^2^ with the feed flow rate of 30 mL/min and 0.25 W/m^2^ with the feed flow rate of 120 mL/min. The power density value of 0.2 W/m^2^ is obtained with the flow rate of 120 mL/min with PECH-H membrane, 0.3 W/m^2^ power density value is achieved with NEOSEPTA AMX and NEOSEPTA CMX membranes with the flow rate of 120 mL/min [30]. In this study, higher power density values are obtained because of the higher IEC of the ER4-BR1.07 membranes. In Altıok et al., RED experiments were performed with Fujifilm Type 2 CEM and Fujifilm Type 2 AEM with three membrane pairs and the salinity ratio of 1:30, a feed flow velocity of 30 mL/min, and the power density value achieved was 0.668 W/m^2^. In the same study, the power density value was found as 0.314 W/m^2^ with three membrane pairs and the salinity ratio of 1:30, flow velocity of 120 mL/min [42]. Altıok et al. carried out RED experiments with RALEX CMX and RALEX AMX membranes with seven membrane pairs, a 1:30 salinity ratio, and a flow rate of 120 mL/min obtaining a power density of 0.213 W/m^2^ [42]. In the same study, a 0.205 W/m^2^ power density was found with seven membrane pairs, 1:30 salinity ratio, and a flow rate of 30 mL/min. Guler et al. performed RED experiments with five membrane pairs of RALEX AMH-PES and RALEX CMH-PES membranes with a flow rate of 30 mL/min and a power density value of 0.5 W/m^2^ was achieved. This power density value is higher than this study because of the number of membrane pairs [11].

## 4. Conclusions

In this study, eco-friendly AEMs were manufactured employing poly(epidochlorohydrin) (PECH) polymer, avoiding the toxic chloromethylation step. Membrane properties, such as area resistance, electrical conductivity, SD, and IEC of these PECH membranes were investigated for the excess diamine ratios of 1, 1.62, 2 and 4 units and blend ratios of 1.07, 1.23, 1.5, and 2. The optimum properties of AEMs were obtained with ER4-BR1.07 membrane with the highest IEC (3.470 mmol/g dry membrane), the highest FCD (5.250 mmol/ g H_2_O), the lowest SD (66%), and highest electrical conductivity (6.443 mS/cm). Testing the synthesized membranes for saline gradient energy recovery in a RED stack, the performance of ER4-BR1.07 AEMs coupled with NEOSEPTA CMX CEMs exhibited the highest power density (0.376 W/m^2^) at the highest flow rate. This work shows the ease of tuning membrane properties by varying the casting solution composition, which opens new room for the development of tailor-made membranes specifically designed for reverse electrodialysis.

## Figures and Tables

**Table 1:** Excess diamine ratio and blend ratio employed in experiments.

Sample Code	Excess Diamine Ratio (ER)	Blend Ratio (BR)
ER1-BR1.07	1.00	1.07
ER1.62-BR1.07	1.62	1.07
ER2-BR1.07	2.00	1.07
ER4-BR1.07	4.00	1.07
ER1.62-BR1.23	1.62	1.23
ER1.62-BR1.5	1.62	1.50
ER1.62-BR2	1.62	2.00

**Table 2:** Technical information of RED stack.

Parameter	Property
Active membrane/electrode area	10 × 10 cm
Number of membranes	3 membrane pairs
Electrodes (Anode and cathode)	Ti/Ru alloy mesh type

**Table 3:** Characteristics of NEOSEPTA CMX membranes [31].

Membranes	Neosepta CMX
Membrane thickness (µm)	181 ± 2
Ion exchange capacity (mmol/g)	1.64 ± 0.01
Permselectivity (%)	92.5 ± 0.6
Swelling degree (%)	21.5 ± 0.2
Area resistance (Ω·cm^2^)	3.43 ± 0.16

**Table 4:** Comparison of the results of this study with the literature data.

Membrane	Thickness (µm)	ER-BR	SD (%)	IEC (mmol/g-Dry Membrane)	FCD (mmol/g H_2_O)	Refs.
ER1.62-BR1.23	132	1.62-1.23	119	2.32	1.95	This study
ER1.62-BR1.07	135	1.62-1.07	110	2.18	1.98	This study
ER2-BR1.07	132	2-1.07	88	3.16	3.59	This study
ER4-BR1.07	139	4-1.07	66	3.47	5.25	This study
PECH B-2	77	4.2-0.333	49	1.68	3.40	[12]
ER4.2-BR1.04	77	4.2-1.04	120	2.80	1.46	[12]
AEM	77	5.5-3.33	30	1.40	4.68	[30]
PECH-H	120–160	2.0-0.6	20.88	2.02	9.70	[34]

ER: Excess diamine ratio; BR: Blend ratio; SD: Swelling degree; IEC: Ion exchange capacity; FCD: Fixed charged density.

**Table 5:** Area resistance and electrical conductivity values of AEMs.

Membrane	Area Resistance (Ω·cm^2^)	Thickness (µm)	Electrical Conductivity (mS/cm)
ER1.62-BR1.07	3.78 ± 0.16	134	3.55 ± 0.16
ER1.62-BR1.23	3.48 ± 0.49	115	3.31 ± 0.49
ER1.62-BR1.5	3.12 ± 0.10	149	4.77 ± 0.10
ER1.62-BR2.0	2.85 ± 0.25	80	2.81 ± 0.25
ER1-BR1.07	3.45 ± 0.19	155	4.50 ± 0.19
ER2-BR1.07	3.83 ± 0.74	138	3.60 ± 0.73
ER4-BR1.07	3.24 ± 0.10	209	6.44 ± 0.10

**Table 6:** The properties of small- and large-scale membranes.

Membrane	Membrane Structure	Thickness (µm)	IEC (mmol/g-Dry Membrane)	SD (%)
ER4-BR1.07 (Dia.: 55 mm)	Diamine ratio:4, Blend ratio:1.07	140	3.47	66
ER4-BR1.07 (MS: 15 cm × 15 cm)		250	3.42	90

Dia.: Petri dish diameter; MS: Membrane size.

**Table 7:** Operational parameters for RED tests.

Parameter	Condition
Number of membrane pairs	3
Flow rates of feed solutions (mL/min)	30, 75, 120
Flow rate of electrode solution (mL/min)	300
Concentration of the feed solutions (g/L)	Dilute NaCl solution: 1 Concentrated NaCl solution: 30
Salinity ratio (g/L low saline solution: g/L high saline solution)	1:30
Electrode solution	0.25 M NaCl; 0.05 M K_3_Fe(CN)_6_; 0.05 M K_4_Fe(CN)_6_
AEMs and CEMs	ER4-BR1.07; NEOSEPTA CMX

**Table 8:** RED performance of the synthesized membranes.

Membrane	Volumetric Flow Rate (mL/min)	Open Circuit Voltage (V)	Maximum Power Density (W/m^2^)
ER4-BR1.07-NEOSEPTA CMX	30	0.395	0.313
	75	0.422	0.336
	120	0.431	0.376

## Data Availability

The data available in this study are available on request from the corresponding author.
